# Microarray meta-analysis to explore abiotic stress-specific gene expression patterns in Arabidopsis

**DOI:** 10.1186/s40529-017-0176-8

**Published:** 2017-05-16

**Authors:** Po-chih Shen, Ai-ling Hour, Li-yu Daisy Liu

**Affiliations:** 1 Biometrics Division, Department of Agronomy, National Taiwan University, Taipei, Taiwan; 2 Department of Life Science, Fu-Jen Catholic University, Xinbei, Taiwan

**Keywords:** Abiotic stress, Gene module, The coefficient of intrinsic dependence, Analysis of variance, The weighted gene co-expression network analysis, Singular value decomposition, Biplot

## Abstract

**Background:**

Abiotic stresses are the major limiting factors that affect plant growth, development, yield and final quality. Deciphering the underlying mechanisms of plants’ adaptations to stresses using few datasets might overlook the different aspects of stress tolerance in plants, which might be simultaneously and consequently operated in the system. Fortunately, the accumulated microarray expression data offer an opportunity to infer abiotic stress-specific gene expression patterns through meta-analysis. In this study, we propose to combine microarray gene expression data under control, cold, drought, heat, and salt conditions and determined modules (gene sets) of genes highly associated with each other according to the observed expression data.

**Results:**

By analyzing the expression variations of the Eigen genes from different conditions, we had identified two, three, and five gene modules as cold-, heat-, and salt-specific modules, respectively. Most of the cold- or heat-specific modules were differentially expressed to a particular degree in shoot samples, while most of the salt-specific modules were differentially expressed to a particular degree in root samples. A gene ontology (GO) analysis on the stress-specific modules suggested that the gene modules exclusively enriched stress-related GO terms and that different genes under the same GO terms may be alternatively disturbed in different conditions. The gene regulatory events for two genes, *DREB1A* and *DEAR1*, in the cold-specific gene module had also been validated, as evidenced through the literature search.

**Conclusions:**

Our protocols study the specificity of the gene modules that were specifically activated under a particular type of abiotic stress. The biplot can also assist to visualize the stress-specific gene modules. In conclusion, our approach has the potential to further elucidate mechanisms in plants and beneficial for future experiments design under different abiotic stresses.

**Electronic supplementary material:**

The online version of this article (doi:10.1186/s40529-017-0176-8) contains supplementary material, which is available to authorized users.

## Background

Facing the challenge of climate change, raising crop production to feed enough people indicates to increase the tolerance of plants to severe environments (Ronald 2011). Plant is a sessile organism and must maintain a complex system of genetic expression to accommodate the impacts of different environments in order to survive with success (Trewavas 2003). When the plant is subjected to a stress, genes coded on its DNA usually take the initial actions required to trigger proper self-defensive mechanisms (Sachs and Ho 1986). It is therefore straightforward to monitor gene expression patterns and their interactions as the first step to deciphering the underlying mechanisms of a plant subjected to stresses. Embraced by rapidly developed biotechnologies, it has become very convenient to accurately monitor global gene expression under different circumstances in living organisms (Ritchie et al. 2015). Although the sequencing of messenger RNA by the latest generation of sequencing technology (also known as RNA-Sequencing or RNA-Seq) is more straightforward, sensitive, and accurate in terms of the quantification of gene expressions, the systematic error rates and costs of said technology remain high compared to those of microarray technology, which has been in use for more than two decades (Mantione et al. 2014).

After collecting a global set of gene expressions, finding differentially expressed genes is the first step in deciphering the underlying mechanisms of a plant that copes with stress. In addition, biologists have recently been asking more about the systematic explanations of gene expression patterns. (e.g., Atkinson and Urwin 2012; Hahn et al. 2013; Priest et al. 2014). Such inquiries have motivated the advancement of gene set analysis and the utilization of microarray data to make inferences regarding genetic networks. Gene set analysis concerns the disturbed gene sets instead of individual genes whereas the gene sets of interest are predetermined (e.g., the co-expressed genes, the genes in the same category of the gene ontology, the genes involved in the same metabolic pathway, etc.) (Kaever et al. 2014, 2015; Rest et al. 2016). Network inference, which is the focus of this study, links genes with edges that indicate potential associations to depict the possible interactions among the chosen set of genes (Todaka et al. 2012; Rasmussen et al. 2013; Nakashima et al. 2014).

The construction of a correlation network is one approach that can be taken after a microarray experiment, while conducting a weighted gene co-expression network analysis (WGCNA) is another. The former can be accomplished by computing the pairwise Pearson correlation coefficients of genes and connecting a given gene pair if the Pearson correlation coefficient exceeds a user-specified threshold (Song et al. 2012). The choice of the threshold, however, might be very subjective, and different thresholds result in networks with different topologies (Borate et al. 2009). A WGCNA, however, preserves all possible edges in the network but assigns different weights to them instead. By clustering the genes with high-weight edges, WGCNA is able to determine the modules (gene sets) of genes highly associated with each other according to the observed expression data. In this study, we further extend the application of WGCNA in three directions:

1. Instead of using the correlation coefficient as the measure of the association between genes, we choose the coefficient of intrinsic dependence (CID).
2. We explore stress-specific modules by conducting the analysis of variance on the expression levels of the Eigen genes representing the gene modules and by comparing the edge weights of the stress-specific modules.
3. A biplot is introduced to visualize the stress-specific modules for convenience in further interpretation.

Along with long-term development of the technology, a huge amount of results from a wide range of microarray experiments has been accumulated. As of August 18, 2016, data from a total of 179 microarray experiments had been included in the Arabidopsis Information Resource (TAIR) database. Typical experiments have consisted of several treated samples under a particular condition and several controlled samples as the background of comparison for the reasons of purification and simplicity. However, analyzing the expression patterns under one stressed condition versus those under control conditions can only reveal a corner of a huge puzzle. One is not able to depict an overview of the entire system or of the interactions between the impacts caused by different stress sources on the living organisms. Therefore, in this study, we combine all possible samples from different stress conditions and perform a meta-analysis of the gene regulatory network on the combined dataset.

To that end, the coefficient of intrinsic dependence (CID), instead of the typical Pearson correlation coefficient, is used to measure the association between genes because the Pearson correlation coefficient only measures the linearity of the gene associations. However, past studies (Liu 2005; Liu et al. 2009) have shown that a nonlinear relationship between the expressions of two associated genes might occur in some cases. The CID does not require distributional and functional assumptions regarding the data and is useful for analyzing noisy microarray data. Relatedly, while systematic errors are well controlled in highly developed microarray technology, samples from different experiments contribute noise to each other when a meta-analysis is conducted due to the fact that the expression patterns from different experiments have a wide range of variation (Ramasamy et al. 2008; Campain and Yang 2010). The CID had been applied to investigate gene regulatory events incorporating the Galton–Pearson correlation coefficient (Liu et al. 2009, 2012), to identify associations among multivariate variables (Liu and Tsai 2013), and to select relevant features on a step-by-step basis according to their importance in relation to the target variable (Hsiao and Liu 2016). In this study, we strictly followed the definition and methodology of CID described in Hsiao and Liu (2016) and focus on utilizing the CID in measuring the magnitude of association in general between genes based on microarray gene expression data.

A CID matrix is used to construct the weighted gene co-expression network produced by a WGCNA. According to the weighted network, gene modules containing genes with similar expression patterns are then identified. The WGCNA further performs a principle component analysis (Pearson 1901; Hotelling 1933; Zhang and Horvath 2005) on the expression matrix of each gene module and uses the first principle component (designated as “Eigen gene”) as the representative of the gene module. The expression of the Eigen gene is the linear combination of the expressions from all genes in the gene module, which has been utilized to identify quantitative-trait-associated gene modules by computing the Pearson correlation coefficient between the observed values of the quantitative trait and the expression levels of the Eigen gene (Zhang and Horvath 2005; Langfelder and Horvath 2008). In this study, it is the qualitative variable (i.e., the treated conditions of the sample) that is of interest. We propose to utilize the analysis of variance for identification of stress-related gene modules by using the expression levels of the Eigen gene as the dependent variable and the treatment conditions of the sample as the independent variable.

**Fig. 1:**
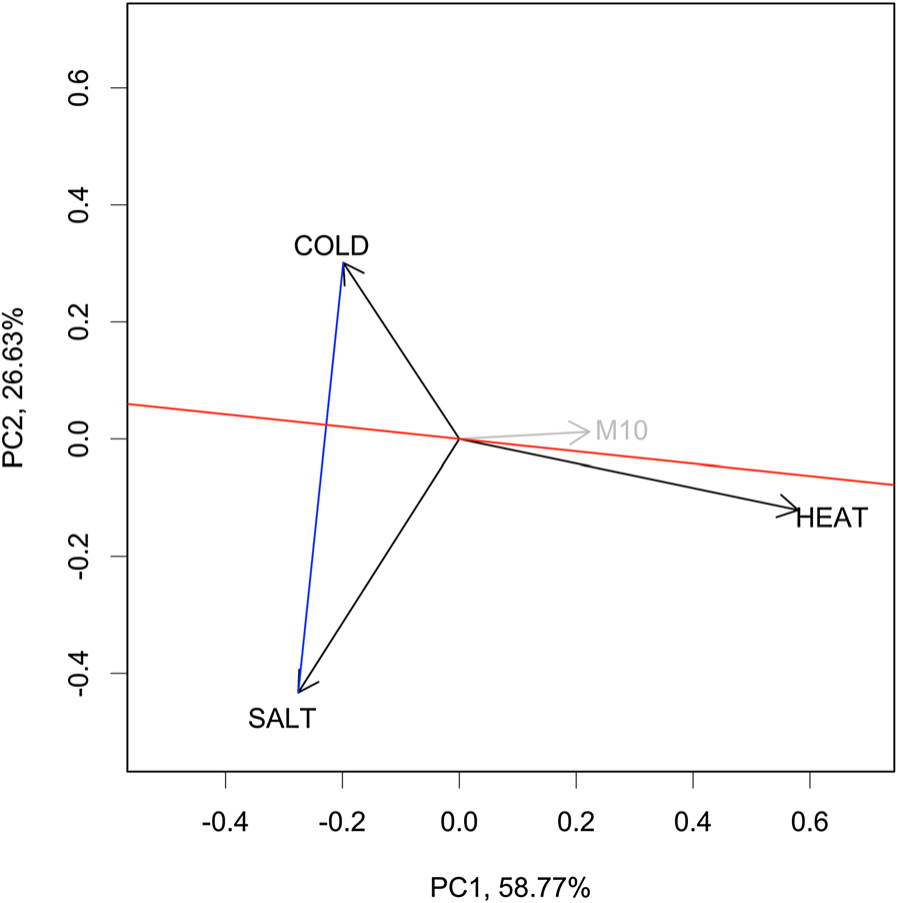
An illustration of a biplot. The *blue line* represents the connection between two stresses, and the *red line* passes through the origin and runs perpendicular to the* blue line*. The *gray* and *black arrows* are vectors of each gene module and stress, respectively

Finally, we present the stress-related gene module using a biplot (Fig. 1), which unifies the information from both the gene module (shown as a gray arrow) and treatment conditions (shown as black arrows) in a single two-dimension plot. The biplot was introduced by Gabriel (1971); Yan and Kang (2002) subsequently described various methods for visualizing and interpreting a biplot. In our case, the length and direction of the projection of a specified condition vector on the vector of the gene module represents the magnitude of the impact of that condition on the gene module. A biplot can also assist in the interpretation of stress-specific modules. For example, an illustration of a biplot shown in Fig. 1, gene module M10 is up-regulated under the heat condition (as indicated by the acute angle between the M10 and HEAT vectors) but down-regulated under the cold and salt conditions (as indicated by the obtuse angles between the M10 and the COLD and SALT vectors). In addition, M10 is heat-specific, but it cannot tell the difference between the cold and salt conditions (the M10 vector is close to the red line, which is perpendicular to the line connecting two condition points; see Materials and Methods).

## Methods

The microarray expression data were downloaded from TAIR database (Huala et al. 2001). Our dataset consisted of 24 cold-treated, 24 salt-treated, 28 drought-treated, 16 heat-treated, and 36 controlled time-series arrays (Additional file 1: Table S1). All 128 arrays were hybridized using the Affymetrix ATH1 chip, which contains 22,810 probe sets (genes). The downloaded microarray raw data was preprocessed using the robust multi-array average (RMA) method and log2 transformed by RMAExpress version 1.0.5 (Bolstad et al. 2003; Irizarry et al. 2003a, b). The output of RMAExpress was taken log2 again to make the expression data more normally distributed (Additional file 2: Figure S1) in order to fulfill the essential assumption of following the analysis of variance. The processed microarray data were subjected to the analyses of variance (ANOVA) to identify the genes differentially expressed using the design models:

$${\text{R}}_{\text{ijkl}} = \upmu_{\text{Ri}} + {\upalpha}_{\text{Rij}} + {\upalpha \uptau}_{\text{Rijk}} + \upepsilon_{\text{Rijkl}} \quad {\text{for}}\;{\text{samples}}\;{\text{of}}\;{\text{root}}\;{\text{tissues}},\;{\text{or}}$$

$${\text{S}}_{\text{ijkl}} = \upmu_{\text{Si}} + \upalpha_{\text{Sij}} + \upalpha \uptau_{\text{Sijk}} + \upepsilon_{\text{Sijkl}} \quad {\text{for}}\;{\text{samples}}\;{\text{of}}\;{\text{shoot}}\;{\text{tissues}},$$

where R_ijkl_ (S_ijkl_) is the normalized expression level of the lth replicate for the ith gene under the jth condition at kth time point in the root (shoot) samples; μ_Ri_ (μ_Si_) is the overall average expression level for the ith gene in the root (shoot) samples; α_Rj_ (α_Sj_) is the condition effect observed in the root (shoot) samples; ατ_Rjk_ (ατ_Sjk_) is the time effect under the jth condition in the root (shoot) samples; and ε_Rijkl_ (ε_Sijkl_) is the sampling error. The analysis of variance which we performed to determine a differential expression gene considered the alternative hypothesis that the gene’s expression was significantly different in at least one of the cold, heat, drought, salted, and controlled conditions. Four p values (two for testing the conditional effects and the other two for testing time effects under different conditions) can be obtained from the above model for each gene. Combining the two p values from the root samples and the two p values from the shoot samples, the smallest one was designated as the overall p value of a gene. The most significant 2281 genes yielding the smallest 10% of overall p values were claimed to be differentially expressed in this study.

The following steps to construct the weighted gene co-expression network were analogue to those of the well-known WGCNA package in R (Langfelder and Horvath 2008). The pairwise CID values for the expression levels of g DE genes were computed to form the g*g CID matrix, C = {c_i,j_}, where c_i,j_ = CID(g_i_|g_j_) for two DE genes, g_i_ and g_j_, by setting the subgroup size of g_j_ to be 20 (Liu et al. 2009). Unlike the correlation matrix, the CID matrix is asymmetric; that means c_i,j_ is not necessarily equal to c_j,i_. The g*g adjacency matrix, A = {a_i,j_}, was set as C taken to the power of three to make the connectivity of the DE genes follow the scale-free property of the biological network (Albert 2005; Pavlopoulos et al. 2011). Specifically, in this study, the connectivity of gene g_i_ for the asymmetric adjacency matrix was defined as Σ_j_a_i,j_ = Σ_j≠i_(c_i,j_)^3^, i,j = 1, …, 2281. The adjacency matrix, A, was analyzed by WGCNA with minModuleSize = 60 to obtain the gene modules (Langfelder and Horvath 2008).

The Eigen gene of each gene module was defined as the first principle component of the genes in the module (Langfelder and Horvath 2008). We performed the singular value decomposition (SVD) toward the s by m expression matrix M = {m_ij_}, where m_ij_ is the average expression level of the Eigen gene of jth (j = 1, …, m) module under the ith (i = 1, …, s) condition. More specifically, the SVD decomposed the matrix M into

$${\mathbf{M}} = {\mathbf{USV}}^{T} = \left( {{\mathbf{US}}^{{{1 \mathord{\left/ {\vphantom {1 2}} \right. \kern-0pt} 2}}} } \right)\left( {{\mathbf{VS}}^{{{1 \mathord{\left/ {\vphantom {1 2}} \right. \kern-0pt} 2}}} } \right)^{T} = {\mathbf{U}}^{*} {\mathbf{V}}^{*T}$$

where **U** and **V** are s by r left and m by r right singular matrices, respectively (Golub and Van Loan 1996), **S** is an r by r diagonal matrix having the singular values, and r is the number of non-zero values of the diagonal of S. The first two columns of U* and V* are referred as the first two principle components (PCs) that most explain the variability of the numbers in the rows (conditions) and the columns (modules) of **M**, respectively.

By overlaying the values of the first two PCs in U* and V* together in a two-dimension plot (known as the “biplot”), we can visualize the effects of the conditions and the modules and their interactions more easily (Gabriel 1971; Yan and Kang 2002). According to the inner-product property of a biplot (Yan and Kang 2002), the projection of a module vector on a condition vector represents the magnitude of the effect of the gene module on the condition, or vice versa. If the angle between two vectors is less than 90° (greater than 90°), the gene module is up-regulated (down-regulated) in the condition. We further connect two points of two conditions (for example, the blue line in Fig. 1 represents such a connection) on the biplot and draw a straight line passing through the origin and running perpendicular to the line connecting two condition points (for example, the red line in Fig. 1). Any module vector having a small angle to the perpendicular line (the red line in Fig. 1) cannot effectively distinguish the two conditions, meaning that similar expression levels (i.e., the projection lengths) on the gene module have been observed in the two conditions. Therefore, we claim that the gene module is specific for a condition if it fulfills two criteria: (1) the length of the gene module vector projected on the condition vector is relatively long, and (2) the gene module vector is almost perpendicular to the connected line (e.g., the blue line in Fig. 1) between any other two conditions. The genes in the stress specific gene modules were verified through gene ontology (GO) analysis using agriGO analytic tools (Du et al. 2010).

## Results

*Arabidopsis thaliana* (Arabidopsis) microarray expression data consisting of 22,810 probe sets (genes) and 216 samples under five conditions (control, cold, drought, heat, and salt) were analyzed. The data were first preprocessed in order to fulfill the normality assumption of ANOVA (Additional file 2: Figure S1), which were run separately on the root and shoot samples. The most significant 2281 genes under different conditions were collected for further analysis. We had observed the tissue-specific responses of the genes under different conditions; only 554 genes were included in the top 10% lists for both the root and shoot samples. Furthermore, there were 813 genes that were differentially expressed only in the shoot samples and 914 genes that were differentially expressed only in the root samples.

**Fig. 2:**
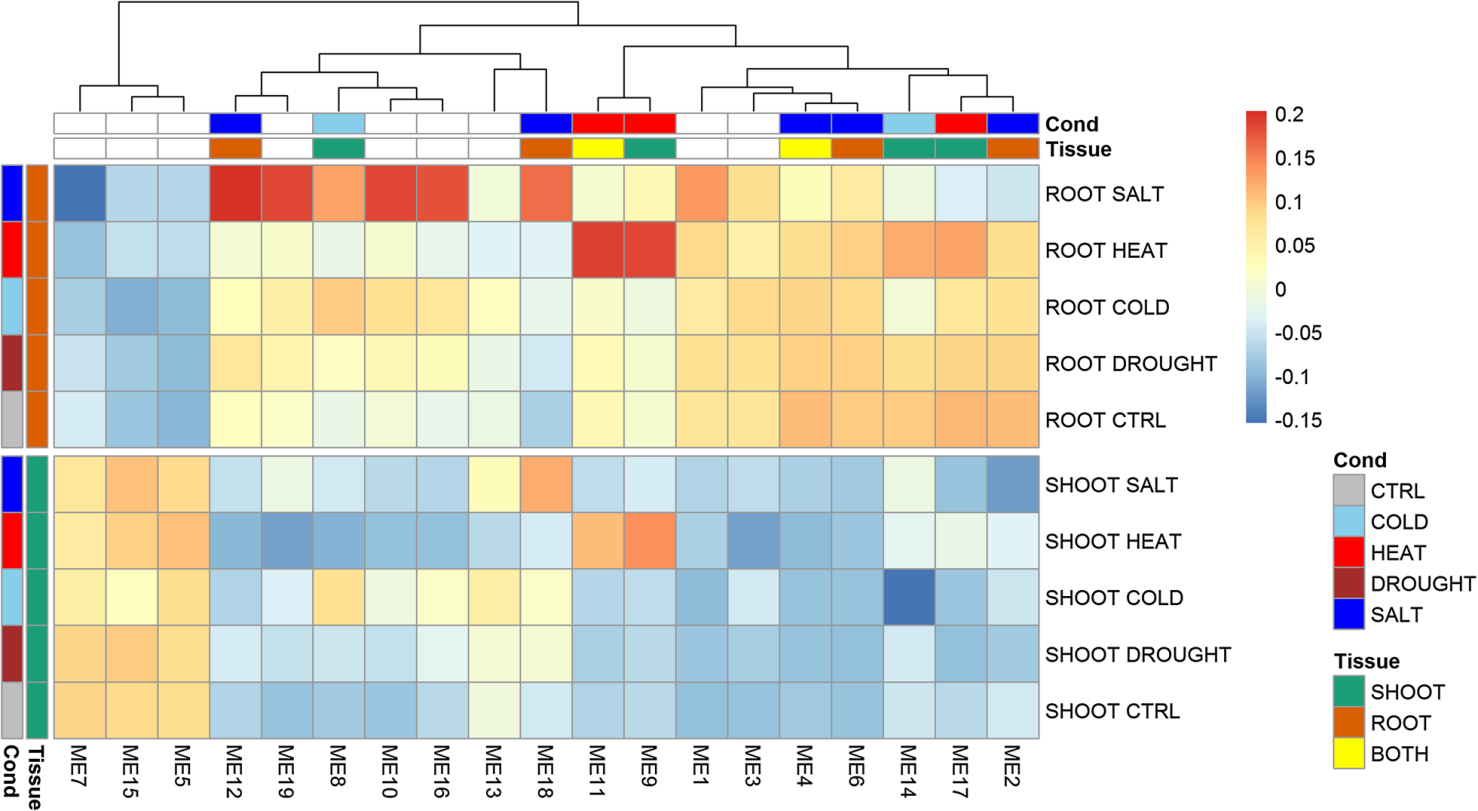
A heatmap consisting of 19 gene modules, 5 stress conditions, and 2 tissue types. This heatmap was consisting of 19 gene modules (M1 to M19), 5 stress conditions (cold, heat, salt, drought, and control), and 2 tissue types (root and shoot tissues)

From the 2281 DE genes, the WGCNA resulted in 19 gene modules (Additional file 3: Table S4). The sizes of the gene modules ranged from 60 to 295 genes, with an average of 120.05 genes (Additional file 4: Table S2). The Eigen gene (ME) for each gene module was designated as the first principle component after conducting the singular value decomposition (SVD) on the expression matrix of the gene module. Figure 2 shows the patterns of average expressions for 19 Eigen genes of all conditions in shoot/root samples. Fourteen out of the 19 Eigen genes (ME1-ME4, ME6, ME8-ME12, ME14, ME16-ME17, and ME19) were mostly highly expressed in root samples. Three Eigen genes (ME5, ME7, and ME15) were expressed more in shoot samples, but the other two Eigen genes (ME13 and ME18) had similar patterns of expression in both root and shoot samples.

**Fig. 3:**
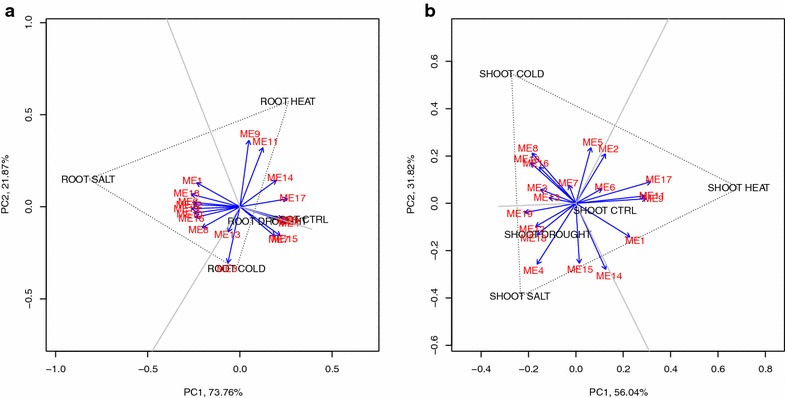
Biplots of 19 Eigen genes and 5 stress conditions. Those biplots were consisting of 19 Eigen genes (ME1 to ME19) and 5 stress conditions (cold, heat, salt, drought, and control) for **a** root tissues, and **b** shoot tissues, respectively

By showing both the diversities of the Eigen genes and the experiments in one biplot, we aimed to link the expression patterns and the abiotic stresses to find the so-called tissue-specific or abiotic stress-specific modules. To simplify the biplot, we used arrows to present the Eigen gene vectors and text to indicate the ends of the abiotic-stress vectors in different tissues starting from the origins (Fig. 3). The SALT and HEAT vectors are the longest among those of all five conditions in the root and shoot samples, respectively, indicating that the expressions of the Eigen genes have relatively large variation under the salt and heat conditions in the root and shoot samples, respectively. The DROUGHT vectors in both the root and shoot samples were very short, meaning that the expressions of the Eigen genes were close to their average values under the drought condition, which can also be observed through the heatmap (Fig. 2). Also, the DROUGHT vectors were very close to the CTRL vectors (the two vectors in root/shoot samples pointed in the same direction and were almost parallel to each other). This means that the expression patterns from the drought-treated samples were very similar to those from the control samples (Fig. 2). Therefore, the drought condition was ignored when searching for stress-specific modules.

## Discussion

By examining the angles between the Eigen gene vectors and the condition vectors in root or shoot samples, we identified the abiotic stress-responsive modules. A smaller angle implies a stronger interaction between the Eigen gene and the condition. We subjectively selected the Eigen genes having angles of less than 15° to any condition vector for further inspection (italics in Tables 1, 2). Under the cold condition, five of the Eigen gene vectors (ME3 and ME9 in the root samples and ME7, ME8, and ME14 in the shoot samples) had angles of less than 15° to the cold vectors; six Eigen gene vectors (ME3, ME11, and ME13 in the root samples and ME9, ME11, ME17, and ME19 in the shoot samples) had angles of less than 15° to the heat vectors. For salt vectors, six Eigen gene vectors (ME2, ME4, ME5, ME6, ME12, and ME18 in the root samples and only ME2 and ME4 in the shoot samples) had angles of less than 15° to them. Note that most of the salt-responsive Eigen genes were present in the root samples and that there were slightly more temperature- (cold- or heat-) responsive Eigen genes detected in the shoot samples. The expressions of the Eigen genes were also tissue-specific because the responsive modules in root and shoot samples were mostly different for a selected condition.

**Table 1:** Angles between each gene module and stress for root samples

Module	Length	C	H	S	P(C,S)	P(H,S)	P(C,H)
*ME9*	0.364	*−4.444*	17.070	87.317	23.562	−28.981	−80.381
*ME11*	0.344	−17.980	*3.535*	−79.148	*10.026*	−42.517	86.084
*ME3*	0.312	*8.814*	*−12.700*	88.313	−19.192	33.352	84.751
*ME18*	0.273	−78.822	−79.664	*4.051*	−73.172	−54.284	*−2.885*
*ME15*	0.269	57.671	−79.185	−25.201	−85.677	33.134	18.265
*ME1*	0.268	−64.099	85.613	18.774	−87.896	−39.561	*−11.838*
*ME19*	0.264	84.335	−62.821	*12.793*	−56.329	−71.128	−19.728
*ME17*	0.262	−77.256	55.742	−19.871	49.250	78.206	26.807
*ME6*	0.257	−87.773	−70.713	*4.900*	−64.222	−63.235	*−11.836*
*ME4*	0.254	74.502	83.984	*−8.370*	77.492	49.965	*1.434*
*ME2*	0.253	76.637	81.849	*−6.236*	75.357	52.100	*0.700*
*ME12*	0.253	89.584	−68.070	*7.543*	−61.579	−65.878	−14.479
*ME10*	0.251	78.739	−57.225	18.388	−50.733	−76.723	−25.324
ME7	0.248	53.104	−74.618	−29.768	−81.110	28.567	22.832
ME16	0.248	73.152	−51.638	23.975	−45.146	−82.311	−30.911
ME14	0.245	−50.562	29.048	−46.565	22.556	−75.100	53.501
ME5	0.239	77.480	81.006	*−5.393*	74.514	52.943	*1.543*
ME8	0.234	57.615	−36.101	39.512	−29.609	82.153	−46.448
ME13	0.151	21.791	*−0.277*	75.337	*−6.215*	46.328	−82.272

The italic underline gene modules had length greater than 0.25

The italics angles were less than 15 degrees

**Table 2:** Angles between each gene module and stress for shoot samples

Module	Length	C	H	S	P(C,S)	P(H,S)	P(C,H)
*ME17*	0.329	−79.780	*10.677*	−43.221	*13.794*	79.689	47.246
*ME14*	0.306	*−1.953*	71.056	55.047	67.939	*2.044*	−51.022
*ME4*	0.305	−58.792	−52.105	*1.792*	−55.222	58.883	*−5.817*
*ME9*	0.299	−67.399	*1.704*	−55.601	*1.414*	67.308	59.626
*ME11*	0.293	−69.798	*0.695*	−53.203	*3.812*	69.706	57.228
*ME8*	0.281	*14.763*	−54.340	−71.763	−51.223	*−14.672*	67.737
*ME1*	0.269	−31.729	37.374	88.729	34.256	31.638	−84.704
*ME10*	0.255	21.774	−47.329	−78.773	−44.212	−21.683	74.748
*ME15*	0.254	−22.970	−87.927	34.029	88.956	23.062	−30.004
ME2	0.244	57.258	53.639	*−0.258*	56.756	−57.349	*4.283*
ME5	0.244	41.448	69.449	−15.551	72.566	−41.540	*11.526*
ME19	0.220	73.898	*−4.795*	49.102	*−7.912*	−73.807	−53.127
ME16	0.218	18.926	−50.177	−75.925	−47.060	−18.834	71.900
ME18	0.210	−77.204	−33.693	20.204	−36.811	77.295	−24.229
ME12	0.196	−86.352	−24.545	29.353	−27.662	86.443	−33.378
ME3	0.160	42.199	−26.904	80.802	−23.787	−42.107	−84.827
ME6	0.127	86.898	23.999	−29.899	27.116	−86.990	33.924
ME13	0.116	52.040	−17.063	70.961	*−13.946*	−51.948	−74.986
ME7	0.083	*5.531*	−74.634	−51.469	−71.516	*−5.622*	47.444

The italic underline gene modules had length greater than 0.25

The italics angles were less than 15 degrees

We further identified Eigen genes which were specifically regulated only under the cold, heat, or salt condition by drawing reference lines on the biplot. A line connecting the ends of any two vectors of conditions, C_1_ and C_2_, was made; this line was called L(C_1_,C_2_) for convenience. Then we made another line, called P(C_1_,C_2_), passing through the origin and running perpendicular to L(C_1_,C_2_). A “stress-specific” module for the condition C_3_ would be almost parallel to both P(C_1_,C_2_) and the vector of C_3_. The angles between the Eigen gene vectors and all three perpendicular lines, P(C[OLD], S[ALT]), P(H[EAT], S[ALT]), P(C[OLD], H[EAT]) are also shown in Tables 1 and 2. When searching for the stress-specific Eigen genes, their lengths were also taken into consideration to identify the Eigen genes that were more affected by the condition of interest; a shorter Eigen gene vector implied that the expression levels of that Eigen gene did not change much under different conditions. The stress-specific Eigen gene vectors with a length greater than 0.25 are marked in italics and underlining in Tables 1 and 2.

In shoot samples, two Eigen gene vectors (ME8 and ME14) under the cold condition (Fig. 3b) had very sharp angles (less than 15°) to P(HEAT, SALT). This implies that the expression patterns of these two Eigen genes are similar under the heat and salt conditions but very different under the cold condition in shoot samples (Fig. 2). Similarly, ME9 and ME17 were heat-specific Eigen genes only in shoot samples. Four Eigen genes (ME2, ME6, ME12, and ME18) were identified as salt-specific only in root samples. Two Eigen genes, ME4 and ME11, were identified as salt-specific and heat-specific, respectively, in both root and shoot tissues.

**Fig. 4:**
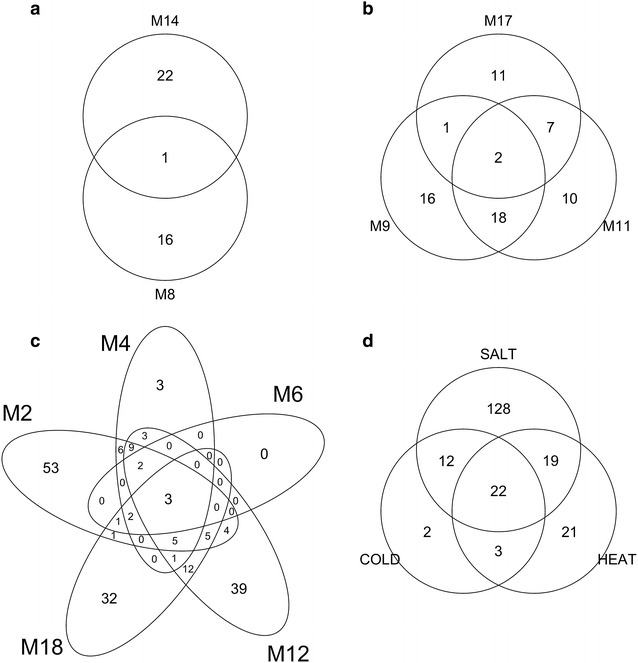
Venn diagrams of enriched gene ontology items. Those venn diagrams were produced for **a** cold-specific, **b** heat-specific, **c** salt-specific, and **d** all stress-specific gene modules. The *numbers* in the venn diagrams were the numbers of enriched GO items in the stress-specific gene modules. For example, in **a**, there were 22, 16, and 1, GO items enriched in M14 only, M8 only, and in both M8 and M14

We validated our findings through gene ontology (GO) enrichment analysis using the genes in the selected gene modules. The lists of the enriched GO are provided in S3 Table. There were 39 GO terms enriched by two cold-specific modules, M8 and M14 (Fig. 4a), with only one cellular component term, “plasma membrane” (GO: 0005886), in common. According to the enriched GO terms, M8 and M14 are complementary to each other and specify different aspects of cold-responsive mechanisms. In particular, the genes in M8 largely participate in the biological processes of stress response (GO: 0006950), temperature stimulus response (GO: 0009266), and cold response (GO: 0009409) (Additional file 5: Table S3A). Cold may also stimulate chemical and endogenous responses in the cells that cause them to accumulate carbohydrate (GO: 0009743) and chitin (GO: 0010200). However, the GO terms enriched by the genes in M14 mostly belong to molecular function and cellular component categories.

Of three heat-specific gene modules, M9 and M17 were specifically identified in shoot samples, while M11 was identified in both root and shoot samples. Similarly, we expected that shoot-only M9 and M17 exclusively enriched heat-related GO terms. The three common GO terms among the 55 enriched in either M9 or M17 were more general GO terms including “response to chemical stimulus” (GO: 0042221), “response to stimulus” (GO: 0050896), and “plasma membrane” (GO: 0005886) (Fig. 4b). Combining the results from three modules, there were several heat-related GO terms that drew our attention, including “response to heat” (GO: 0009408), “protein folding” (GO: 0006457), “response to high light intensity” (GO: 0009644), “response to oxidative stress” (GO: 0006979), “response to radiation” (GO: GO: 0009314), and “response to cadmium ion” (GO: 0046686) (Additional file 5: Table S3B). This implied that the plant may simultaneously suffer adversities from light, radiation, and cadmium toxicity when under heat stress.

Under the salt stress, genes in the M4 module were specifically disturbed in both root and shoot samples. The three GO terms enriched only in M4 were “response to inorganic substance” (GO: 0010035), “response to metal ion” (GO: 0010038), and “response to cadmium ion” (GO: 0046686) (Fig. 4c; Additional file 5: Table S3C). The module M6 contained genes in response to osmotic stress (GO: 0006970), especially salt stress (GO: 0009651). This meant that the enriched GO terms of M6 mostly overlapped with those enriched in the other salt-specific modules. Genes in the M2 module were more involved in the processes of localization (GO: 0051179), cellular component biogenesis (GO: 0044085), macromolecule biosynthesis (GO: 0009059), and vitamin biosynthesis (GO: 0009110). A large portion of genes in the M12 module were expressed in response to wounding (GO: 0009611), biotic stimulus (GO: 0009607), organic acid metabolism (GO: 0006082), and cellular amino acid metabolism (GO: 0006520). The M18 module contains genes related to reproduction (GO: 00000003), fruit and seed development (GO: 0010154 and GO: 0048316), and embryonic/post-embryonic development (GO: 0009790 and GO: 0009791).

**Fig. 5:**
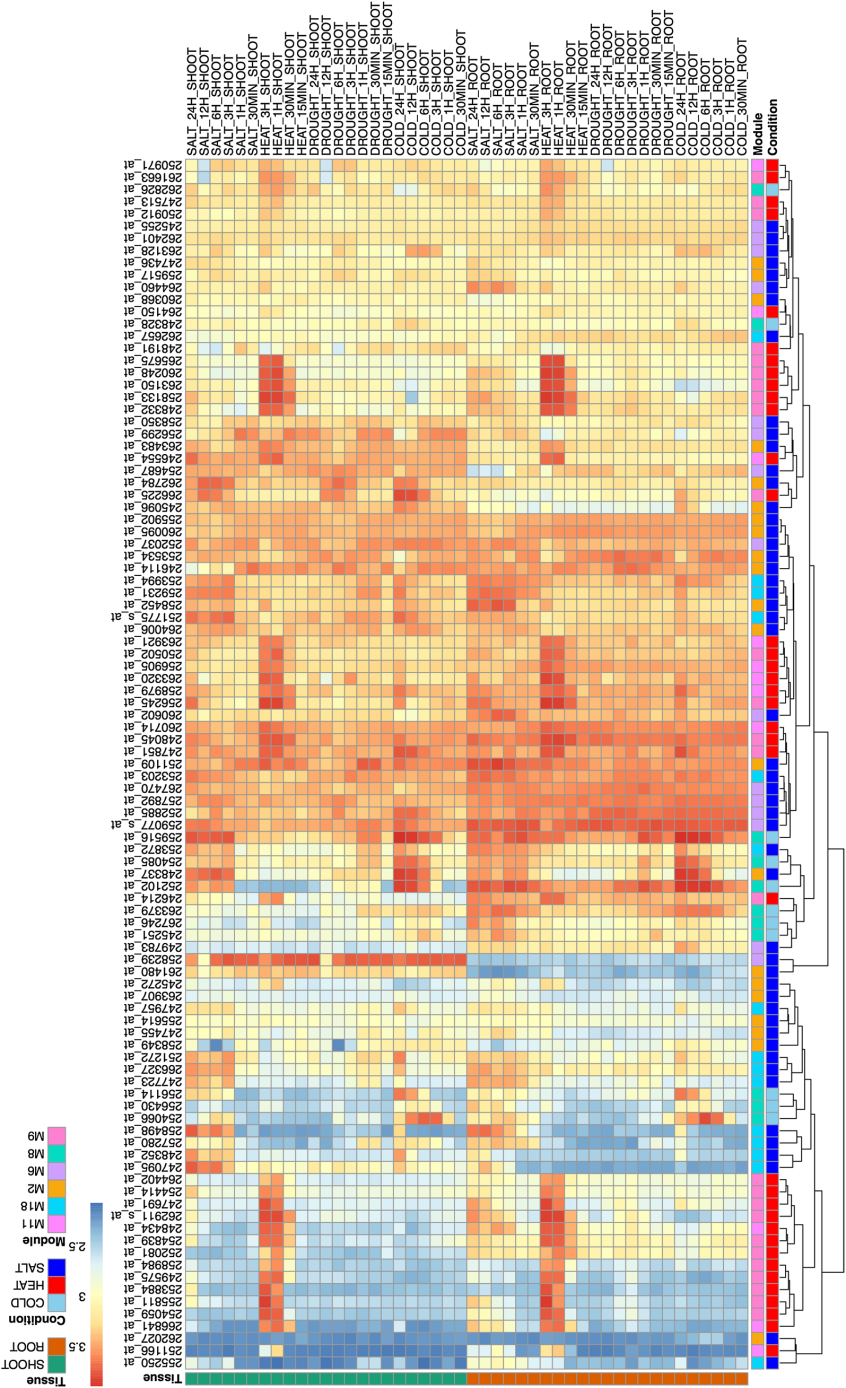
A heatmap of the enriched GO term, “response to abiotic stimulus” (GO: 0009628). This heatmap was produced using the 99 genes corresponding to the enriched GO term, “response to abiotic stimulus” (GO: 0009628) in the four stress conditions

Although the stress-specific modules were composed of different genes, they eventually enriched the same 22 GO terms (Fig. 4d), including “primary metabolic process” (GO: 0044238), “response to chemical stimulus” (GO: 0042221), “response to stimulus” (GO: 0050896), “response to temperature stimulus” (GO: 0009266), “response to stress” (GO: 0006950), “response to hormone stimulus” (GO: 0009725), “response to endogenous stimulus” (GO: 0009719), and “response to abiotic stimulus” (GO: 0009628) (Additional file 5: Table S3D). That means that different genes in the same GO may be disturbed in different ways under different conditions. For example, the expressions for the 99 genes corresponding to the enriched “response to abiotic stimulus” (GO: 0009628) in different modules presented tissue-specific and/or stress-specific patterns (Fig. 5).

The inferred stress-specific gene regulatory events were partly supported by experimental results through literature search. For instance, we compared the AGI locus identifiers of the 98 genes in the cold-specific gene module M8 to those known as cold-temperature responsive genes in the Gramene Pathway Browser (http://plantreactome.gramene.org/PathwayBrowser/#SPECIES=9079025&DIAGRAM=9085214&PATH=9085019,9085215&DTAB=MT) and only two genes, *DREB1A* (AT4G25480) and *DEAR1* (AT3G50260), were matched. They belong to CBF1 homolog and RAP2 homolog, respectively, in the Gramene pathway modules. *DREB1A* was the intermediate between the well-known cold induced transcription factor, ICE1, and the cold responsive genes, *RD29A* and *COR15A* (Yamaguchi-Shinozaki and Shinozaki 2006), while *DEAR1* would repress the expression of *RD29A* and *COR15A* under the cold stress (Tsutsui et al. 2009).

## Conclusion

In this study, we identified abiotic stress-specific modules after conducting a weighted gene correlation network analysis (WGCNA) using the analysis of variance and biplot visualization. Our first step in doing so was to differentiate the relevant gene module(s) according to different categorical traits (the different stress conditions, in our case) by the analysis of variance, something which might not have been accomplished by an analysis of correlation. Furthermore, the geometric interpretation of a biplot aims to utilize mRNA levels to point out to plant physiologists a plausible direction for further in vitro validation of tissue-specific and/or stress-specific mechanisms. The readers need to be aware of the fact that the heatmaps and biplots presented in this study were constructed based on the expression levels of the “pseudo” Eigen genes, which represent the diverse gene expressions in the gene modules specified by the WGCNA. It is thus possible that none of the genes in a given module exactly matches the expression patterns of the Eigen gene. The results of the analyses only provide hints about the underlying biological processes, which need to be further confirmed, as for example, by the gene ontology analysis in this study. In conclusion, our approach has the potential to further elucidate stress-specific mechanisms in plants via meta-analysis of massive amounts of microarray data. It can be used to complement the conventional bioinformatics analyses associated with the studied phenotypes.

## Additional files

**Additional file 1: Table S1.** A list of arrays using in this study. The dataset consisted of 24 cold-treated, 24 salt-treated, 28 drought-treated, 16 heat-treated, and 36 controlled time-series arrays. All 128 arrays were treated in 2 tissues, root and shoot 50-50.

**Additional file 2: Figure S1.** Boxplots of gene expressions. (A) Raw data. (B) Data after pre-processing using RMA. (C) Data after pre-processing using RMA and log-2 transformed once. (D) Data after pre-processing using RMA and log-2 transformed twice.

**Additional file 3: Table S4.** The list of 2281 significant genes used in this study.

**Additional file 4: Table S2.** The number of genes of each gene module. The number of gene and the probe sets ID of each gene module is listed in this table. The largest gene module was M1 including 295 genes and the smallest one was M19 including 60 genes.

**Additional file 5: Table S3.** Lists of enriched GO terms of each abiotic stress-specific gene module. These tables are shown the enriched GO terms in the (A) cold-specific, (B) heat-specific, (C) salt-specific, and (D) all condition-specific gene modules.

## Abbreviations

GO: gene ontology
RNA-Seq: RNA-sequencing
WGCNA: the weighted gene co-expression network analysis
CID: the coefficient of intrinsic dependence
TAIR: The Arabidopsis Information Resource
RMA: robust multi-array average
ANOVA: analysis of variance
SVD: singular value decomposition
PC: principle component
