# Supplementary figures and images for: Microarray meta-analysis to explore abiotic stress-specific gene expression patterns in Arabidopsis
Source: Bot Stud. 2017 May 16;58:22. doi: 10.1186/s40529-017-0176-8 (PMC5432924; doi:10.1186/s40529-017-0176-8)

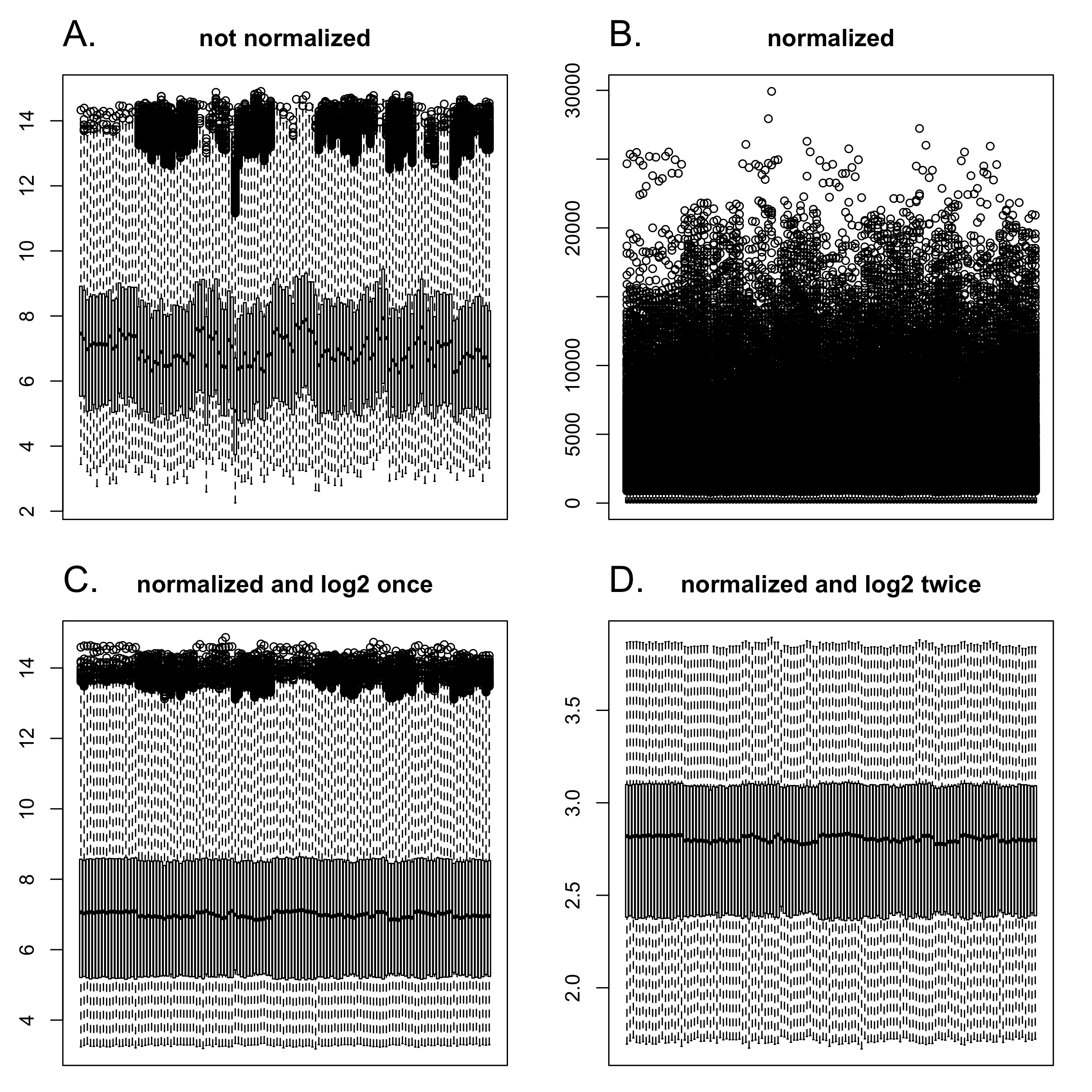

Supplement: Supplementary file 2 — Additional file 2: Figure S1. Boxplots of gene expressions. (A) Raw data. (B) Data after pre-processing using RMA. (C) Data after pre-processing using RMA and log-2 transformed once. (D) Data after pre-processing using RMA and log-2 transformed twice. [file 40529_2017_176_MOESM2_ESM.png]
